# *GOrilla*: a tool for discovery and visualization of enriched GO terms in ranked gene lists

**DOI:** 10.1186/1471-2105-10-48

**Published:** 2009-02-03

**Authors:** Eran Eden, Roy Navon, Israel Steinfeld, Doron Lipson, Zohar Yakhini

**Affiliations:** 1 Molecular Cell Biology Department, Weizmann Institute of Science, Rehovot, Israel; 2 School of Computer Science, Tel Aviv University, Tel Aviv, Israel; 3 Agilent Laboratories, Tel-Aviv, Israel; 4 Computer Science Department, Technion, Haifa, Israel

## Abstract

**Background:**

Since the inception of the GO annotation project, a variety of tools have been developed that support exploring and searching the GO database. In particular, a variety of tools that perform GO enrichment analysis are currently available. Most of these tools require as input a target set of genes and a background set and seek enrichment in the target set compared to the background set. A few tools also exist that support analyzing ranked lists. The latter typically rely on simulations or on union-bound correction for assigning statistical significance to the results.

**Results:**

*GOrilla *is a web-based application that identifies enriched GO terms in ranked lists of genes, without requiring the user to provide explicit target and background sets. This is particularly useful in many typical cases where genomic data may be naturally represented as a ranked list of genes (e.g. by level of expression or of differential expression). *GOrilla *employs a flexible threshold statistical approach to discover GO terms that are significantly enriched at the *top *of a ranked gene list. Building on a complete theoretical characterization of the underlying distribution, called mHG, *GOrilla *computes an exact p-value for the observed enrichment, taking threshold multiple testing into account without the need for simulations. This enables rigorous statistical analysis of thousand of genes and thousands of GO terms in order of seconds. The output of the enrichment analysis is visualized as a hierarchical structure, providing a clear view of the relations between enriched GO terms.

**Conclusion:**

*GOrilla *is an efficient GO analysis tool with unique features that make a useful addition to the existing repertoire of GO enrichment tools. *GOrilla*'s unique features and advantages over other threshold free enrichment tools include rigorous statistics, fast running time and an effective graphical representation. *GOrilla *is publicly available at:

## Background

The availability of functional genomics data has increased dramatically over the last decade, mostly due to the development of high-throughput microarray-based technologies such as expression profiling. Automatic mining of these data for meaningful biological signals requires systematic annotation of genomic elements at different levels. The Gene Ontology (GO) project [1] is a collaborative effort aimed at providing a controlled vocabulary to describe gene product attributes in all organisms. GO consists of three hierarchically structured vocabularies (ontologies) that describe gene products in terms of their associated biological processes, cellular components and molecular functions. The building blocks of GO are terms, the relationship between which can be described by a directed acyclic graph (DAG), a hierarchy in which each gene product may be annotated to one or more terms in each ontology.

Since its inception, many tools have been developed to explore, filter and search the GO database. A comprehensive list of available tools is provided at the Gene Ontology web site . One of the most common applications of the GO vocabulary is *enrichment analysis *– the identification of GO terms that are significantly overrepresented in a given set of genes [2]. Enrichment may suggest possible functional characteristics of the given set. For example, enriched GO terms in a set of genes that are significantly over-expressed in a specific condition may suggest possible mechanisms of regulation that are put into play, or functional pathways that are activated in that condition.

A large repertoire of tools for enrichment analysis has been developed in recent years, including GoMiner [3], FatiGO [4], BiNGO [5], GOAT [6], DAVID [7] and others. In general, these tools accept as input a target set of genes that is compared to a given background set of genes, or to a default "complete" background set. Some subset of GO terms from one or more of the three ontologies is scanned for enrichment in the target set relative to the background set, and terms for which significant enrichment is discovered are reported. The statistical test used for enrichment analysis is typically based on a hypergeometric or binomial model.

The most common form of output is a list of enriched terms. This simple approach allows the user to identify terms that are most significantly enriched but may lose substantial information regarding the relations between these terms. A more informative approach is to present the enrichment results in the context of the DAG structure of the respective ontology. In a typical case, the list of significantly enriched GO terms may include several related terms at varying significance levels. Identifying the clusters of enriched terms in the GO hierarchy becomes much simpler if the DAG structure is made available. A few tools visualize the results of enrichment analysis in the DAG structure, including the downloadable version of GoMiner [3], the CytoScape plug-in BiNGO [5], GOLEM [8], GOEAST [9] and GOTM [10]. A particularly friendly and useful GO enrichment analysis tool is GO::TermFinder which is provided at the Saccharomyces Genome Database (SGD, [11]). This tool provides a color-coded map of the enriched GO terms. It is, however, limited only to analysis of *S. cerevisiae *genes and requires specifying an explicit target set.

In many practical cases, functional genomic information used as the input for the GO enrichment analysis may be naturally represented as a ranked list. For most applications, the requirement for an input target gene set forces the user to set some arbitrary threshold and define the target set as all genes with ranks above (or below) the threshold. For example, genes may be naturally scored and ranked according to their differential expression between two conditions. However, defining the specific set of genes that are differentially expressed requires setting an arbitrary threshold. Unfortunately, the results of the enrichment analysis may often depend on the specific threshold that is set. Tools that use the simple hypergeometric distribution require setting such a fixed threshold.

A few tools have been developed that use a threshold free approach including GSEA [12], FatiScan [13], GO-stat [14], GeneTrail [15] and iGA [16]. The widely used GSEA tool uses a statistic that is similar to Kolmogorov-Smirnov but assigns different weights to the occurrences of genes in different ranks in the list. The tool is not specifically aimed at GO enrichment, and therefore does not offer visualization in terms of the GO DAG structure. In addition GSEA does not provide an exact p-value and estimates the p-value using permutations. The p-values assigned by GSEA are therefore limited by the number of permutations performed. FatiScan is another threshold free tool by the creators of FatiGO. It tests a number of thresholds determined by the user (the default is 30 thresholds) and then corrects for multiple testing using FDR. Again, this tool does not provide an exact p-value. The iGA method uses an iterative approach that circumvents the need for a fixed cutoff by computing the hypergeometric score at all possible cutoffs. iGA does not produce an exact p-value as well. In [17] the authors study the advantages of sample re-sampling. The sample permutation approach is applicable for the analysis of differential gene expression data but not in other applications of gene set enrichment where ranking is inferred otherwise.

As a matter of practicality it is also important for GO enrichment tools to perform in an interactive manner. Many of the existing tools, in particular those based on flexible thresholds that require time consuming simulations, fall short on this desired property.

In this note we describe a web-server interactive software tool, called *GOrilla*, that enables GO enrichment analysis in ranked lists of genes. It is based on previous work [18], in which we describe a statistical framework, called mHG, for enrichment analysis in ranked lists. The method identifies, independently for each GO term, the threshold at which the most significant enrichment is obtained. The significance score is accurately and tightly corrected for threshold multiple testing without the need for time consuming simulations. Consequentially *GOrilla *performs the enrichment analysis on thousands of genes and thousands of GO terms in a few seconds. In the Results section we demonstrate how *GOrilla *can capture relevant biological processes and visualize the results with an easy to use graphical representation of the GO hierarchy, emphasizing on the enriched nodes.

## Implementation

### Enrichment Analysis

A standard approach for identifying enriched GO terms uses the hypergeometric distribution (e.g. [8]). Given a total number of genes *N*, with *B *of these genes associated with a particular GO term and *n *of these genes in the target set, then the probability that *b *or more genes from the target set are associated with the given GO term is given by the hypergeometric tail:

(1) $$\text{Prob}(X\geq b)=\text{HGT}(b;N,B,n)=\sum_{i=b}^{\min(n,B)}\frac{\left(\begin{array}{c} n\\ i \end{array}\right)\left(\begin{array}{c} N-n\\ B-i \end{array}\right)}{\left(\begin{array}{c} N\\ B \end{array}\right)},$$

If a ranked gene list: *g*_1_,...,*g*_*N *_is provided in place of a target set, we define a label vector *λ *= *λ*_1_,...,*λ*_*N *_∈ {0, 1}^*N *^according to the association of the ranked genes to the given GO term, *λ*_*i *_= 1 iff *g*_*i *_is associated with the term. The *minimum hypergeometric *(mHG) score is then defined as:

(2) $$mHG(\lambda )=\underset{1\leq n<N}{\min}HGT(b_{n}(\lambda );N,B,n),$$

where $b_{n}(\lambda )=\sum_{i=1}^{n}\lambda_{i}$. In words, the mHG score is the optimal HGT probability that is found over all possible partitions induced by the gene ranking. As such, this score must be corrected for multiple testing. In previous work we describe a dynamic programming algorithm for computing the exact p-value of a given mHG score [18]. More specifically, given a ranked list of genes, a GO term associated with some of these genes and a corresponding mHG score *s*, the mHG p-value tells us the exact probability of observing an mHG score *s' *≤ *s *under the null assumption that all GO term occurrence configurations in the ranked list are equiprobable. We describe various considerations for efficient implementation of the mHG p-value algorithm elsewhere [19].

### Description of the Tool

*GOrilla *is publicly available as a web-based application at: . The application has two modes of operation:

1. Discovery of enriched GO terms at the top of a ranked list of genes using the mHG statistics (as explained in this paper).

2. Discovery of enriched GO terms in a target set versus a background set and using a hypergeometric model (as commonly done in other applications). In this case the ranking inside these lists is ignored.

**Figure 1:**
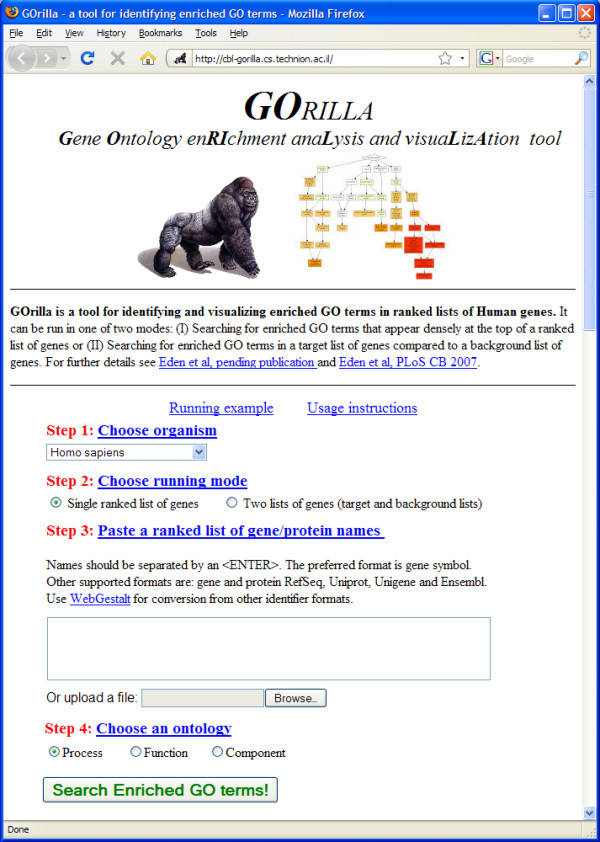
**How to use the *GOrilla *web user interface**. To use the *GOrilla *web interface, the user is required to perform the following four simple steps: (i) choose an organism; (ii) choose a running mode (either flexible threshold or fixed threshold mode) (iii) copy and paste a list (or upload a file) of genes in the case of a flexible threshold or two lists of genes – a target and a background – in the case of a fixed cutoff; (iv) choose an ontology.

The web interface of *GOrilla *is shown in Figure 1. The input to the application is a ranked list or two sets (depending on the mode of operation) that consist of gene names in the following formats: gene symbol, protein RefSeq, Uniprot, Unigene or Ensembl. Gene duplication often occurs in such lists, which can lead to statistically biased results. *GOrilla *automatically removes duplicates keeping the highest ranking occurrence. This includes dealing with duplicates that hide behind different nomenclatures. *GOrilla *currently supports the following organisms: human, mouse, rat, yeast, *D. melanogaster, C. elegans *and *Arabidopsis thaliana*.

**Figure 2:**
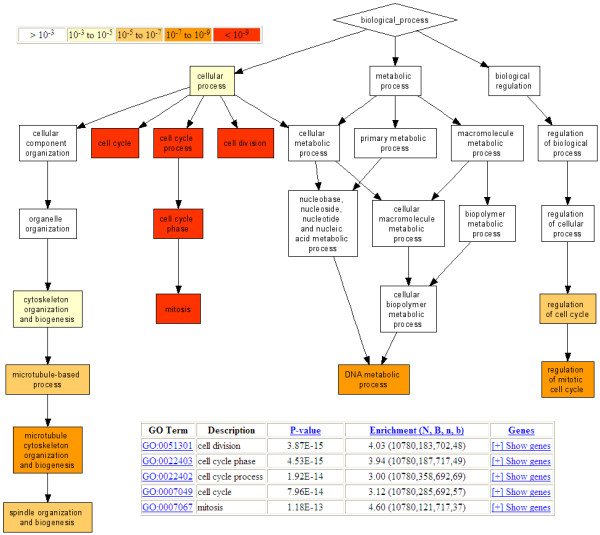
**An example of the *GOrilla *analysis output**. 14,565 genes from the van't Veer dataset were ranked according to their differential expression and given as input to *GOrilla*. The resulting enriched GO terms are visualized using a DAG graphical representation with color coding reflecting their degree of enrichment. Nodes in the graph are clickable and give additional information on the GO terms and genes attributing to the enrichment. N is the total number of genes; B is the total number of genes associated with a specific GO term; n is the flexible cutoff, i.e. the automatically determined number of genes in the 'target set' and b is the number of genes in the 'target set' that are associated with a specific GO term. Enrichment is defined as (b/n)/(B/N).

The output consists of a color-coded trimmed DAG of all significantly enriched GO terms. The output also includes a table consisting of the enriched GO terms (with web links to additional information), the enrichment p-values (computed under the null model as described above) and the relevant annotated genes. The nodes of the resulting DAG, which represent GO terms, are color-coded according to the significance of the detected enrichment and clickable, leading to the relevant entry in the above table. Results can be exported to Excel. Figure 2 depicts an example of the result of enrichment analysis, where the relations between enriched terms can be observed. To generate the GO DAG visualization, *GOrilla *employs AT&T's GraphViz tool [20].

## Results and discussion

To test the performance of *GOrilla *we used the van't Veer et al. breast cancer dataset [21], which is a landmark study in clinical use of gene expression data. This dataset consists of expression profiles containing 14,565 genes measured on 77 breast cancer patients. Different patients showed various prognostic characteristics, 44 with more than 5 years survival versus 33 patients with less than 5 years survival. All genes were ranked according to how well they differentiate between the two groups using a simple t-test. The top of the list contained the genes that were the best separators between the two groups. The ranked list was given as input to *GOrilla *with default running parameters. The result is shown in Fig 2 and highlights a unique set of enriched GO terms that were identified at different cutoffs. The enriched GO terms include mitosis (*p *< 10^-12^, top 717 genes), cell cycle (*p *< 10^-13^, top 692 genes) and microtubule cytoskeleton organization and biogenesis (*p *< 10^-8^, top 927 genes). These enriched GO terms are attributed to genes that were over expressed in patients with bad prognosis and under-expressed in patients with good prognosis, which is in accordance with biological common sense and supports their relevance. The total analysis running time was less than 10 seconds.

A comparison to other web-based tools was performed on the same dataset using each software's default parameters. Web-based tools have several advantages over standalone tools [2] and therefore we only compare *GOrilla *to other web-based tools. The flexible threshold tools Fatiscan [13] and GO-stat [14] were given the ranked list as input while the fixed threshold methods GOEAST and DAVID were given a target set containing all the genes with t-test *p *< 10^-3^, which includes the top 124 genes, and a background set containing the rest of the genes. We note that the *p *< 10^-3 ^choice of fixed threshold for cutting the data, which is often used for this type of tasks, is inherently arbitrary.

A summary of the comparison between the different tools is given in Table 1. DAVID and GOEAST which use a fixed threshold based approach, yielded a similar set of enriched GO terms to the ones yielded by *GOrilla*. Most of these terms were identified with less significant enrichment scores presumably because these methods use an arbitrary cutoff. The flexible cutoff based methods Fatiscan did not identify any enriched GO term while the GO-stat flexible method identified GO terms similar to the ones obtained by *GOrilla*. Running time of different tools varied considerably ranging from a few minutes up to 30 minutes per single analysis, which may be partially due to Monte-Carlo simulations employed by some of the methods for assessing the p-value.

**Table 1:** A comparison of web-based GO enrichment tools.

**Tool**	**P-value and statistical method**	**Flexible threshold**	**Graphical visualization**	**Multiple organisms**	**Running time**
GOrilla	Exact mHG p-value computation (no need for simulations)	+	+	+	7 Sec

Fatiscan [13]	Fischer Exact (FDR corrected for number of thresholds)	+ (predetermined steps of 30)	-	+	30 Min

GO-stat [14]	Wilcoxon Rank-Sum/Kolmogorov Smirnov	+	-	+	2 Min

GOEAST [9]	Hypergeometric	-	+	+	20 Min

SGD [11]	Hypergeometric	-	+	- (only yeast)	2 Min

DAVID [7]	Modified Fischer Exact	-	-	+	2 Min

GOTM [10]	Hypergeometric	-	+	+	2 Min

GoMiner [3]	Fisher Exact	-	- (only in the downloadable version)	+	7 Min

Different GO enrichment tools employ a wide range of statistics and yield different performances. The main features of five different web-based tools are compared to *GOrilla*. To enable a fair comparison all tools were used using default parameters via their web interfaces and applied on the van't Veer dataset. One exception is the SGD tool that only runs on yeast data and was therefore tested on a set of 543 yeast genes and the default background for running time characterization. The *GOrilla *running time for this yeast dataset was also 7 seconds. The running time was measured for the entire analysis, including uploading files and getting the results.

## Conclusion

The main contribution of the application presented herein are:

1. Most other approaches to GO enrichment analysis assume that a target set and a background set are given or evident to the process. It is often the case, however, that genomic data which is the subject of the enrichment analysis step, is naturally represented as a ranked list of elements. *GOrilla *uses a statistical model that supports the discovery of GO terms that are enriched at the top of a ranked list, enabling a threshold to be determined in a data driven manner. It also provides an exact p-value for the observed event, which is not the case for most other available flexible threshold tools.

2. *GOrilla *provides a simple and informative graphical representation of the significantly enriched terms, in the context of the complete DAG representation of the ontology used in the analysis. This graphical representation is color coded based on the p-value attained for each GO term.

3. The application is highly interactive with running time of a few seconds per analysis. This is achieved by using an efficient algorithm for computing the exact mHG p-value, which circumvents the need for simulations, and an efficient software implementation.

## Availability and requirements

Project name: *GOrilla*

Project home page: 

Operating system(s): (Platform independent) web-based application

Programming language: Java

## Authors' contributions

EE co-developed the methods, wrote the software application and led the writing of the manuscript. RN wrote the software application and contributed to the writing of the manuscript. IS wrote the software application and contributed to the writing of the manuscript. DL co-developed the methods, wrote the software application and contributed to writing the manuscript. ZY co-developed the methods, advised on design and features and supervised the study and the writing of the manuscript.
